# Supplementary material for: Nonlinear exposure-response associations of daytime, nighttime, and day-night compound heatwaves with mortality amid climate change
Source: Nat Commun. 2025 Jan 14;16:635. doi: 10.1038/s41467-025-56067-7 (PMC11729900; doi:10.1038/s41467-025-56067-7)
Supplement: Supplementary file 1 — Supplementary Information [file 41467_2025_56067_MOESM1_ESM.pdf]

Supplementary Information

Nonlinear exposure-response associations of daytime, nighttime, and day-night compound heatwaves with mortality amid climate change

Jiangdong Liu[1], Ho Kim[2], Masahiro Hashizume[3], Whanhee Lee[4], Yasushi Honda[5,6], Satbyul Estella Kim[5,6], Cheng He [7], Haidong Kan[1,8,*], Renjie Chen[1,*]

[1] School of Public Health, Shanghai Institute of Infectious Disease and Biosecurity, Key Lab of Public Health Safety of the Ministry of Education and NHC Key Lab of Health Technology Assessment, Fudan University, Shanghai, China
[2] Department of Biostatistics and Epidemiology, Graduate School of Public Health, Seoul National University, Seoul, Republic of Korea
[3] Department of Global Health Policy, Graduate School of Medicine, The University of Tokyo, Tokyo, Japan
[4] School of Biomedical Convergence Engineering, Pusan National University, Yangsan, South Korea
[5] Center for Climate Change Adaptation, National Institute for Environmental Studies, Tsukuba, Japan
[6] Faculty of Health and Sport Sciences, University of Tsukuba, Tsukuba, Japan
[7] Institute of Epidemiology, Helmholtz Zentrum München—German Research Center for Environmental Health (GmbH), Neuherberg, Germany
[8] Children's Hospital of Fudan University, National Center for Children's Health, Shanghai, China

[*] **Correspondence:**
Dr. Renjie Chen, School of Public Health, Fudan University, 130 Dong-An Road, Shanghai 200032, China; E-mail: chenrenjie@fudan.edu.cn;
Dr. Haidong Kan, School of Public Health, Fudan University, 130 Dong-An Road, Shanghai 200032, China; E-mail: kanh@fudan.edu.cn.

The PDF file includes:

Supplementary Method
Supplementary Figs. 1–7
Supplementary Tables 1–5

Supplementary Method:

The description of main statistical models.

The city-specific model is represented by supplementary equations (1) to (2):

$$Y_{it} \sim \textit{quasi-Poisson}(\mu_{it}) \quad (1)$$

$$\mathrm{Log}(\mu_{it}) = \alpha_i + NS(\mathrm{HW}_{i,t}, \mathrm{lag}=6) + NS(\mathrm{RH}_{i,t}) + NS(\mathrm{Date}_{i,t}) + \mathrm{Year}_{i,t} + \mathrm{DOW}_{i,t} \quad (2)$$

where Y_{it} represents the number of deaths on day t in city i, and α is the city-specific intercept. The three types of heatwaves ($HW_{i,t}$) were incorporated as predictors using *quasi*-dummy variables, where non-heatwave days serve as the reference category. The values within each heatwave type are assigned using the Cumulative Excess Heatwave Index (CEHWI). Their lag effects were modeled by a natural cubic spline (NS) with 3 degrees of freedom (*dfs*), with a maximum lag of up to a week (0–6 days) to capture cumulative lagged effects. $NS(RH_{i,t})$ refers to a natural cubic spline of the relative humidity with three *dfs*. $NS(Date_{i,t})$ denotes a natural cubic spline with four *dfs* for day of the season and $Year_{i,t}$ represents a indicator variable for year to account for long-term trends. Day of the week ($Dow_{i,t}$) was included as a categorical variable.

In the second stage, meta-analysis was conducted to pool city-specific estimates. The choice between random-effects and fixed-effects models was based on Cochran's Q test and the I^2 statistic, which quantified the proportion of variability due to the true differences across cities. When the Q test was statistically significant and/or $I^2 \geq 50$, the random effect model based on the residual maximum likelihood estimation would be applied. Otherwise, the fixed effect model would be used. The mixed effects meta-regression model is specified as supplementary equation (3):

$$\hat{\beta}_i = \beta_0 + X_i\beta_i + \eta_i + \varepsilon_i \quad (3)$$

where $\hat{\beta}_i$ is the estimated effect of heatwaves in city i, β_0 is the overall mean effect across cities; X_i is a matrix of predictors in city i; η_i is a random effect of the city-specific deviation; ε_i is the random error of the sampling variability.

The attributable fractions (AFs) of heatwave-related mortality were calculated as supplementary equation (4):

$$AF_{x,t} = \left(1 - \exp(-\sum_{l=l_0}^{L} \beta_{x_{t-l},l})\right) \times 100\% \quad (4)$$

where the formula is consistent with the typical configuration of the regression model applied to analyze the data. $\beta_{x,\ell}$ is the coefficient of relative risk on day t, associated with lagged exposures to CEHWI from $t-\ell$ *(i.e.,* $x_{t-\ell}$*)*, with ℓ ranging from the minimum (ℓ_0) and maximum (L) lags (i.e., lag 0 to lag 6). To estimate the empirical confidence intervals (eCIs) of the AFs, we performed Monte Carlo simulations based on the assumption of a multivariate normal distribution for the point estimate and covariance matrix derived from the regression models. By simulating 1000 random samples, we obtained the 2.5th and 97.5th percentiles of the resultant distributions, which were interpreted as the 95% eCIs.

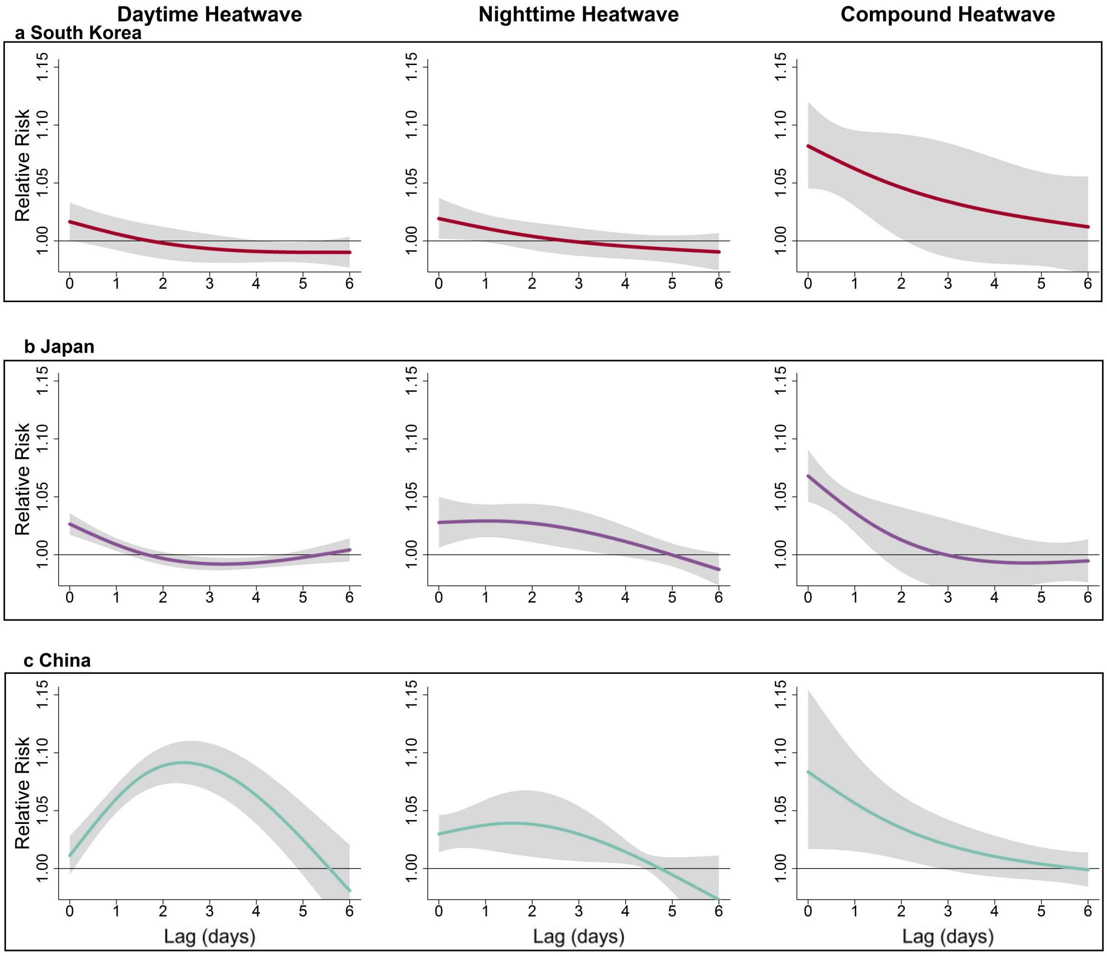

Supplementary Fig. 1. Lag structures for the associations of non-accidental mortality with daytime, nighttime, and day-night compound heatwaves in three countries of East Asia. a, South Korea; b, Japan; c, China. The solid lines represent the estimated relative risks (point estimates) of mortality for each lag day associated with specific heatwave types compared to non-heatwave days, while the shaded areas indicate the corresponding 95% confidence intervals.

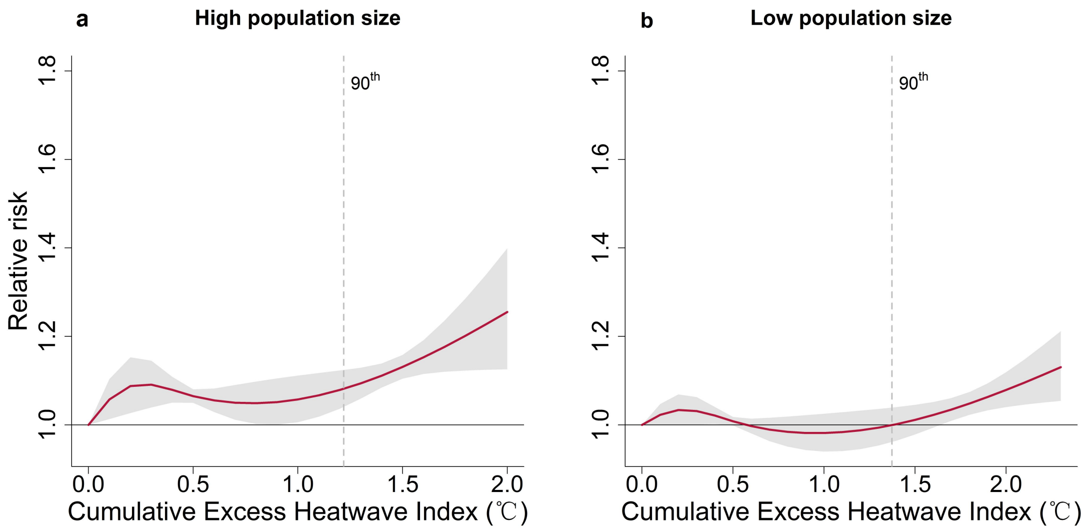

Supplementary Fig. 2. Pooled cumulative exposure-response curves for the associations between daytime heatwaves and non-accidental mortality across different population size levels. a, High population size level; b, Low population size level. The solid lines represent the estimated cumulative relative risks (point estimates) of mortality on heatwave days compared to non-heatwave days, while the shaded areas indicate the corresponding 95% confidence intervals.

Notes: Population size levels were categorized based on the median population size across all cities included in this study (3.3 million residents).

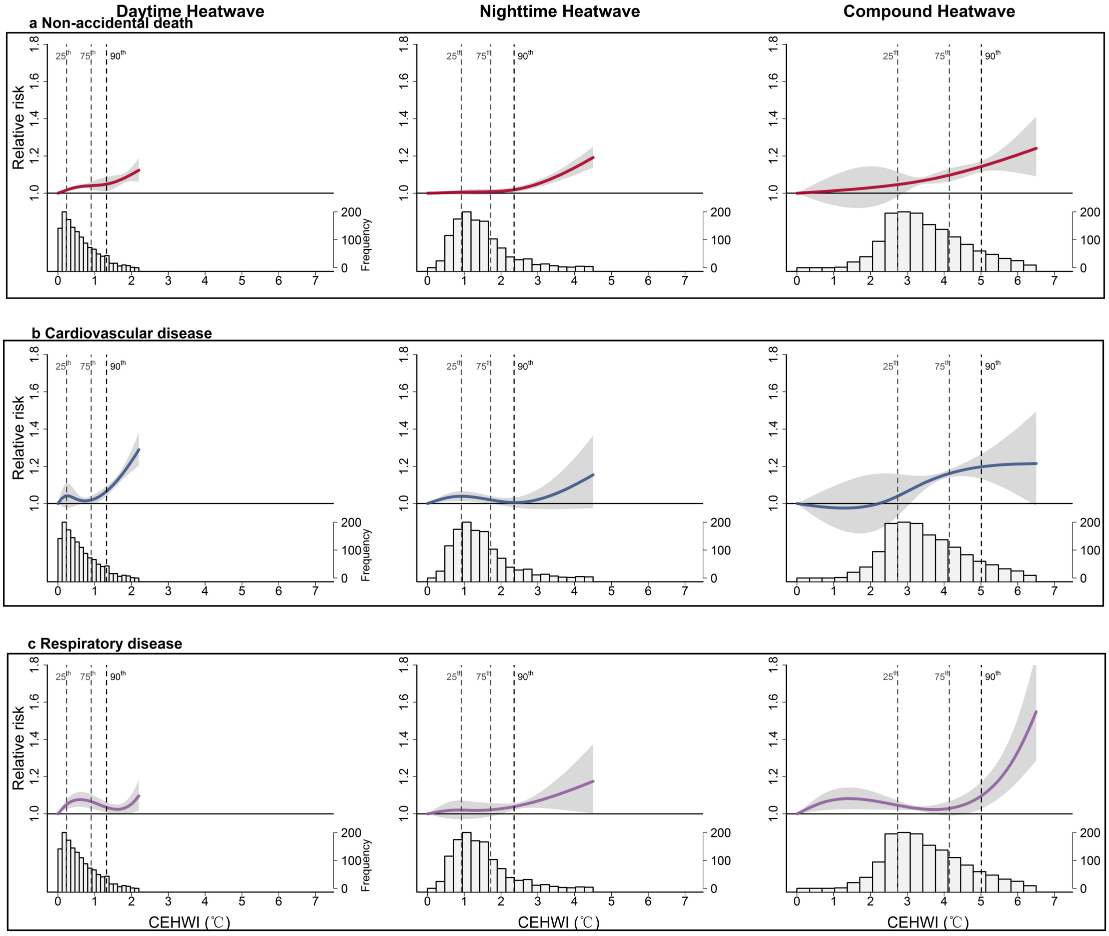

Supplementary Fig. 3. Pooled exposure-response relationship curves of cumulative excess heatwave index (CEHWI) for daytime, nighttime, and day-night compound heatwaves with mortality in East Asia when controlling **PM_{10}**. a, Non-accidental death; b, Cardiovascular disease; c, Respiratory disease. The solid lines represent the estimated cumulative relative risks (point estimates) of mortality on heatwave days compared to non-heatwave days, and the shaded areas represent the corresponding 95% confidence intervals. The vertical dotted lines from left to right indicate the 25th, 75th, and 90th percentiles of the cumulative excess temperature distribution of heatwaves, respectively.

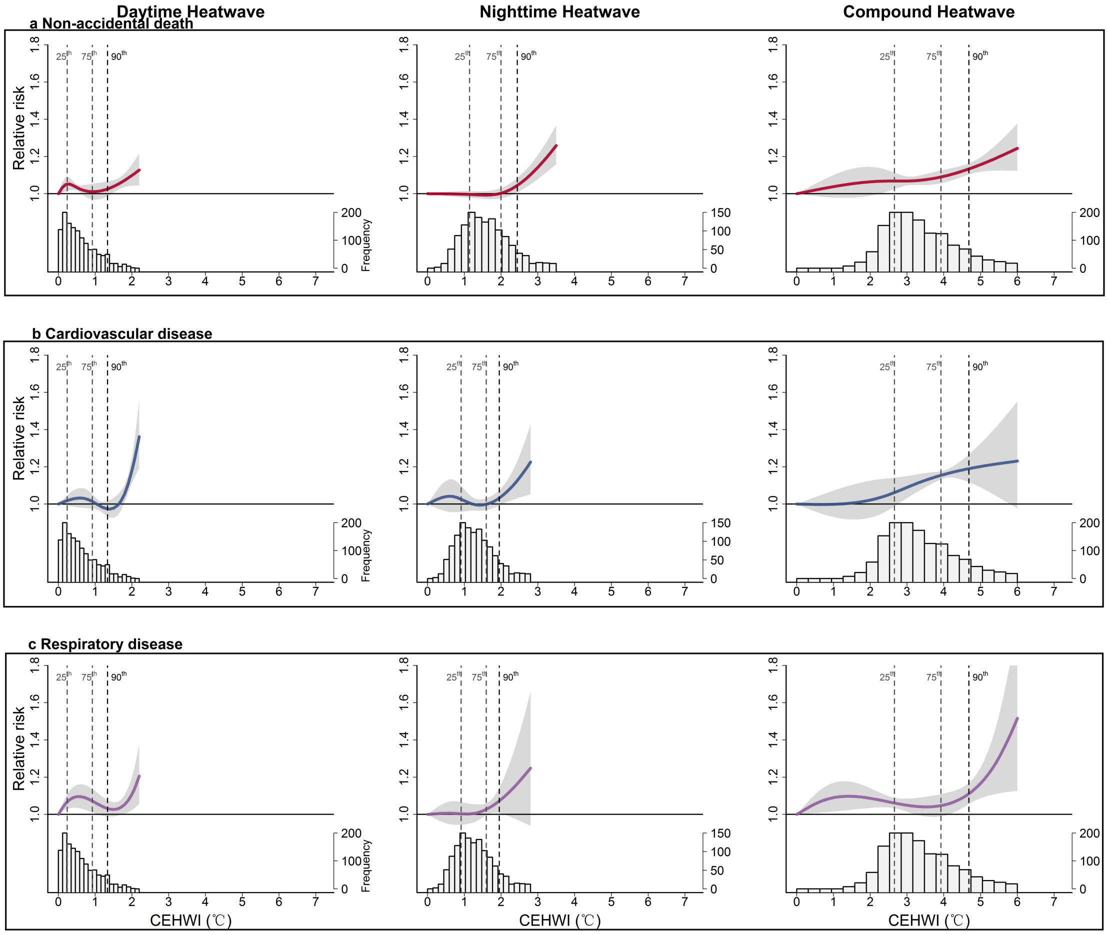

Supplementary Fig. 4. Pooled exposure-response relationship curves of cumulative excess heatwave index (CEHWI) for daytime, nighttime, and day-night compound heatwaves with mortality in East Asia when controlling **O_3**. a, Non-accidental death; b, Cardiovascular disease; c, Respiratory disease. The solid lines represent the estimated cumulative relative risks (point estimates) of mortality on heatwave days compared to non-heatwave days, and the shaded areas represent the corresponding 95% confidence intervals. The vertical dotted lines from left to right indicate the 25th, 75th, and 90th percentiles of the cumulative excess temperature distribution of heatwaves, respectively.

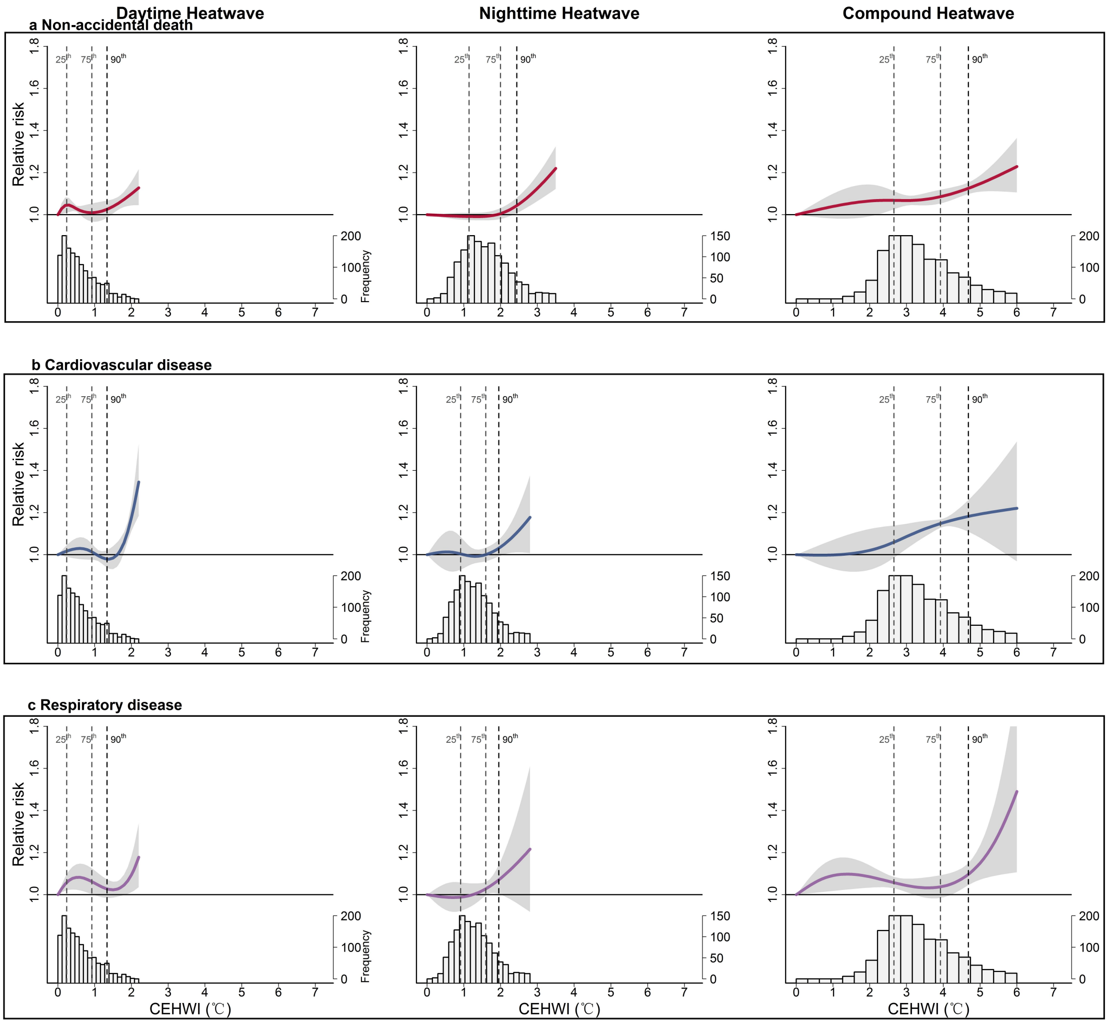

Supplementary Fig. 5. Pooled exposure-response relationship curves of cumulative excess heatwave index (CEHWI) for daytime, nighttime, and day-night compound heatwaves with mortality in East Asia when controlling **PM_{10} and O_3**. a, Non-accidental death; b, Cardiovascular disease; c, Respiratory disease. The solid lines represent the estimated cumulative relative risks (point estimates) of mortality on heatwave days compared to non-heatwave days, and the shaded areas represent the corresponding 95% confidence intervals. The vertical dotted lines from left to right indicate the 25th, 75th, and 90th percentiles of the cumulative excess temperature distribution of heatwaves, respectively.

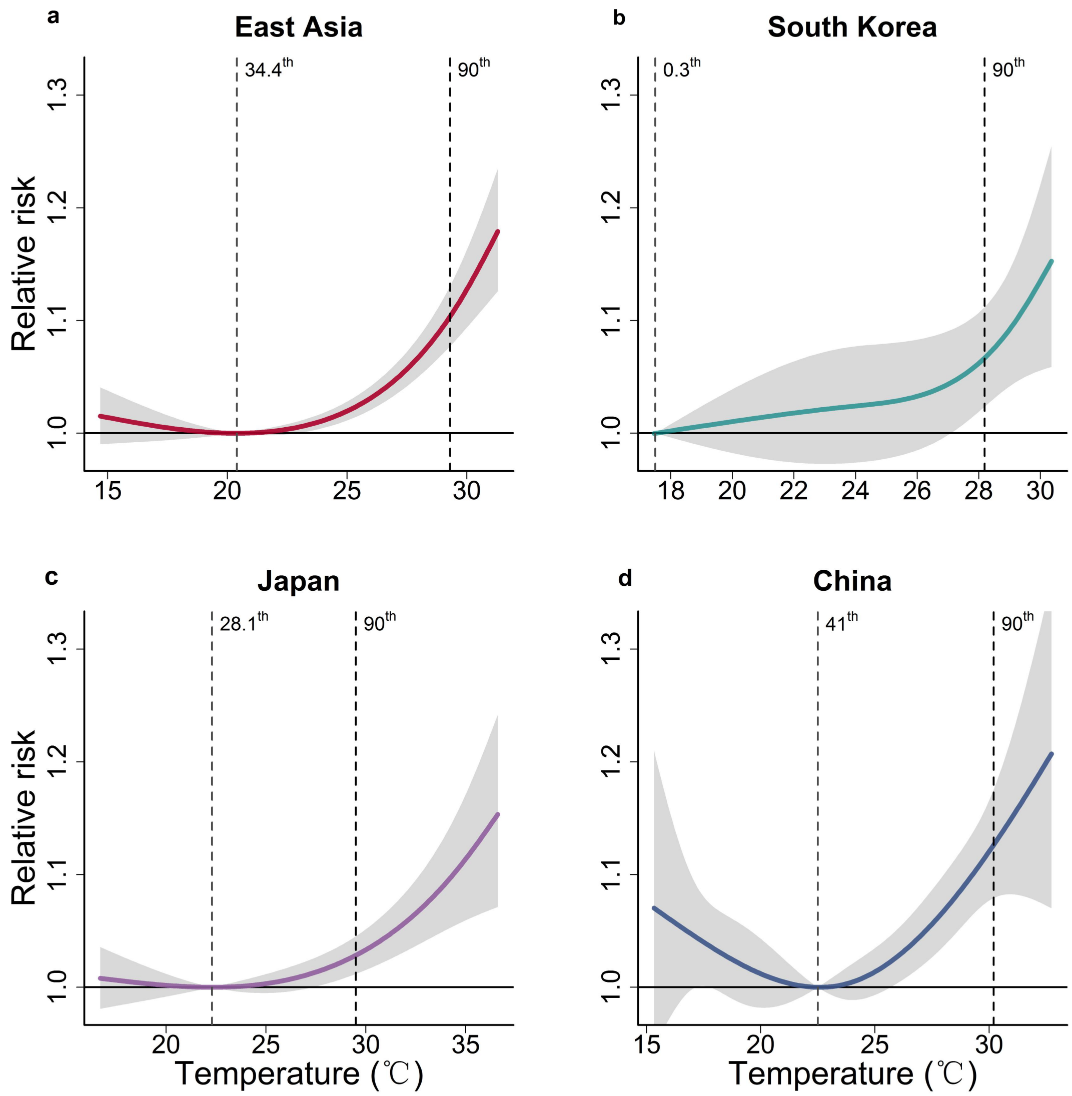

Supplementary Fig. 6. Cumulative exposure-response curves for associations between summer daily average temperature (°C) and non-accidental mortality. a, East Asia; b, South Korea; c, Japan; d, China. The vertical dashed line refers to the minimum mortality temperatures (MMT) and the temperature corresponding to the 90% percentile of local temperature distribution, respectively. The solid lines represent the estimated cumulative relative risks (point estimates) of mortality at a specific temperature compared to the MMT, with the shaded areas indicating the corresponding 95% confidence intervals.

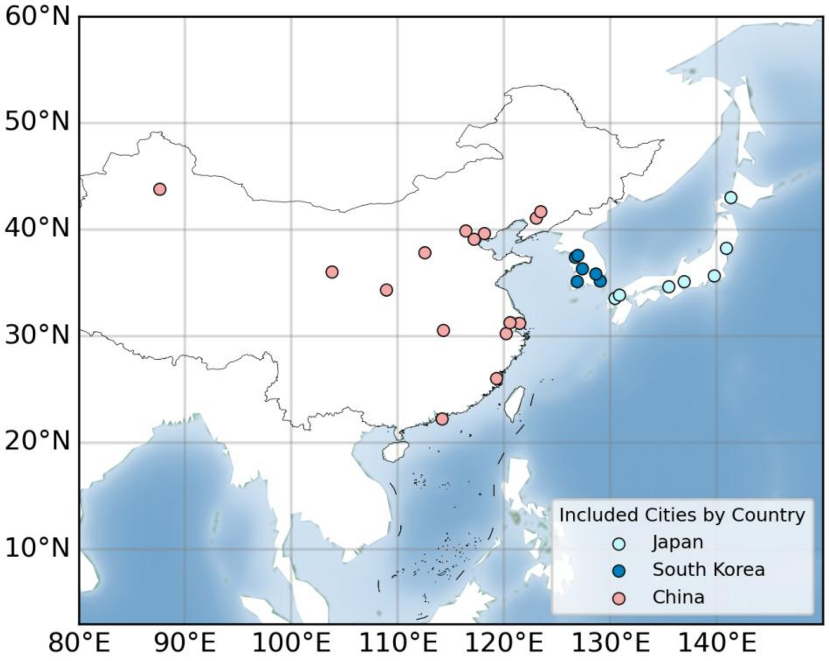

Supplementary Fig. 7. Spatial distribution of the 28 cities included in this study. Seven cities in Japan, six cities in South Korea, and fifteen cities in China.

Supplementary Table 1. Overview of the baseline temperature and heatwave condition and mortality data from the 28 cities during the summer season.

Cities	Period#	Numbers of death			Temp	Numbers of heatwave##		
		NAD	CVD	RD	(℃)	DHW	NHW	CHW
South Korea								
Seoul	1992-2010	171,878	40,442	8,446	24.40	3.05	4.32	2.21
Busan	1992-2010	80,686	21,960	3,760	23.61	3.42	2.95	2.37
Daegu	1992-2010	49,392	11,367	2,387	25.17	4.95	3.21	2.79
Incheon	1992-2010	46,647	11,490	2,433	23.53	2.63	3.32	1.47
Gwangju	1992-2010	25,586	5,031	1,262	24.82	6.16	5.05	1.53
Daejeon	1992-2010	24,834	5,443	1,289	24.41	5.58	4.63	1.89
Japan								
Sapporo	1981-2010	72,395	21,877	8,782	19.78	2.37	3.43	1.67
Sendai	1981-2010	33,632	10,162	3,753	21.53	2.23	2.43	2.37
Tokyo	1981-2010	391,364	122,306	47,373	25.06	2.00	1.53	3.23
Nagoya	1981-2010	99,650	31,701	11,471	25.60	3.50	2.37	2.13
Osaka	1981-2010	153,055	43,818	19,107	26.56	2.37	1.90	2.20
Kitakyushu	1981-2010	54,917	16,074	6,592	26.09	3.00	2.83	1.97
Fukuoka	1981-2010	50,007	13,356	6,379	26.09	3.47	2.67	1.47
China								
Anshan	2004-2006	7,099	3,423	491	24.97	2.67	1.67	3.00
Beijing	2007-2008	18,889	8,282	2,000	26.09	3.00	1.00	5.50
Fuzhou	2004-2006	4,151	1,729	399	28.44	6.33	2.67	0.00
Hangzhou	2002-2004	4,679	1,552	833	27.61	4.00	0.67	5.33
HongKong	1996-2002	51,316	13,375	10,167	28.59	3.00	9.14	1.86
Lanzhou	2004-2008	7,898	No data	No data	18.85	2.40	7.40	3.20
Shanghai	2001-2004	38,427	13,517	4,008	27.65	2.00	3.00	2.25
Shenyang	2005-2008	22,701	10,291	2,119	22.79	1.75	3.00	2.25
Suzhu	2005-2008	10,844	3,649	1,335	27.76	4.75	1.00	6.75
Taiyuan	2004-2008	10,211	3,615	673	23.41	4.20	3.40	6.00
Tangshan	2006-2008	4,178	1,866	787	25.37	1.67	2.00	0.00
Tianjin	2005-2008	3,548	1,968	206	26.20	1.50	0.50	5.75
Wuhan	2003-2005	13,100	7,082	1,505	28.39	4.00	3.67	7.00
Wulumqi	2006-2007	3,107	728	394	23.38	3.00	3.00	2.00
Xian	2004-2008	10,441	4,513	2,717	24.81	3.00	5.60	6.20

#Only for summer periods (June to August)

##The number of heatwave days per summer season

Notes: DHW=daytime heatwave; NHW=nighttime heatwave; CHW=day-night compound heatwave; NAD=non-accidental death; CVD=cardiovascular disease; RD=respiratory disease. Temp=Summer daily average temperatures.

Supplementary Table 2. The national relative risks and attributable fractions of daily non-accidental, cardiovascular, and respiratory mortality associated with daytime, nighttime, and day-night compound heatwave across East Asia.

**Causes**	**Countries**	**Heatwave**	**AF (%; 95% eCI)**	**RR (95% CI)**
Non-accidental death				
	South Korea	DHW	0.10 (0.01, 0.19)	1.00 (0.94, 1.08)
		NHW	0.01 (0.00, 0.02)	1.00 (0.98, 1.02)
		CHW	0.40 (0.26, 0.51)	1.36 (1.18, 1.57)
	Japan	DHW	0.04 (0.01, 0.07)	0.98 (0.94, 1.04)
		NHW	0.08 (0.04, 0.12)	1.07 (1.04, 1.10)
		CHW	0.22 (0.14, 0.29)	1.12 (1.09, 1.16)
	China	DHW	0.32 (0.23, 0.38)	1.18 (1.11, 1.24)
		NHW	0.04 (0.00, 0.07)	1.00 (0.93, 1.09)
		CHW	0.43 (0.23, 0.54)	1.24 (1.16, 1.32)
Cardiovascular diseases				
	South Korea	DHW	0.07 (-0.12, 0.22)	1.01 (0.95, 1.06)
		NHW	0.01 (-0.03, 0.04)	1.00 (0.95, 1.06)
		CHW	0.39 (0.25, 0.46)	1.38 (1.21, 1.58)
	Japan	DHW	0.23 (0.15, 0.29)	0.99 (0.93, 1.06)
		NHW	0.06 (-0.06, 0.15)	1.07 (1.02, 1.13)
		CHW	0.49 (0.32, 0.60)	1.35 (1.22, 1.51)
	China	DHW	0.28 (0.10, 0.36)	1.10 (1.03, 1.18)
		NHW	0.06 (-0.18, 0.23)	1.04 (0.98, 1.10)
		CHW	0.57 (0.16, 0.75)	1.23 (1.12, 1.34)
Respiratory diseases				
	South Korea	DHW	0.24 (-0.13, 0.51)	1.17 (1.09, 1.26)
		NHW	0.15 (-0.39, 0.48)	1.12 (1.01, 1.24)
		CHW	0.30 (0.01, 0.53)	1.32 (1.08, 1.60)
	Japan	DHW	0.23 (0.08, 0.34)	1.04 (0.99, 1.10)
		NHW	0.10 (0.03, 0.16)	1.05 (1.01, 1.09)
		CHW	0.31 (0.13, 0.45)	1.48 (1.31, 1.67)
	China	DHW	0.20 (-0.22, 0.42)	1.13 (1.05, 1.22)
		NHW	0.06 (-0.06, 0.16)	1.06 (1.00, 1.12)
		CHW	0.25 (-0.02, 0.41)	1.31 (1.13, 1.53)

Notes: AF=attributable fraction; RR=relative risk; DHW=daytime heatwave; NHW=nighttime heatwave; CHW=day-night compound heatwave.

Supplementary Table 3. The exploration of heterogeneity in non-accidental mortality risk associated with daytime, nighttime, and day-night compound heatwave.

Heatwave Type	Predictor	Q-test#	I^2 (%)	Wald test##
**Daytime heatwave**				
	None	0.04	23.4%	No applicable
	T_{mean}	0.05	22.2%	0.19
	T_{mean} Range	0.05	22.6%	0.22
	GDP	0.05	22.3%	0.13
	Climate zone	0.03	28.4%	0.85
	**Population**	0.11	17.0%	**<0.01**
	Latitude	0.03	25.6%	0.77
	Longitude	0.04	24.2%	0.48
	All predictor	0.21	14.6%	No applicable
**Nighttime heatwaves**				
	None	0.04	24.6%	No applicable
	T_{mean}	0.03	27.5%	0.69
	T_{mean} Range	0.03	27.6%	0.87
	GDP	0.03	27.8%	0.91
	Climate zone	0.07	23.0%	0.32
	Population	0.03	27.8%	0.91
	Latitude	0.03	26.9%	0.71
	Longitude	0.03	26.4%	0.88
	All predictor	0.01	39.4%	No applicable
**Compound heatwaves**				
	None	<0.01	64.1%	No applicable
	T_{mean}	<0.01	64.1%	0.52
	T_{mean} Range	<0.01	65.2%	0.57
	GDP	<0.01	64.2%	0.12
	Climate zone	<0.01	64.3%	0.40
	Population	<0.01	61.6%	0.17
	Latitude	<0.01	63.4%	0.16
	Longitude	<0.01	63.9%	0.16
	All predictor	<0.01	61.7%	No applicable

#Q-test was for meta-regression.

##Wald test was for each predictor.

Supplementary Table 4. The pooled attributable fractions of daily non-accidental, cardiovascular, and respiratory mortality associated with daytime, nighttime, and day-night compound heatwave across East Asia, using different modeling setting.

Sensitivity Test	Heatwave	NAD	CVD	RD
Test 1	DHW	0.09 (0.04, 0.12)	0.13 (0.05, 0.18)	0.20 (0.07, 0.28)
	NHW	0.04 (0.00, 0.08)	0.05 (0.01, 0.08)	0.10 (0.03, 0.14)
	CHW	0.30 (0.24, 0.34)	0.46 (0.34, 0.51)	0.27 (0.18, 0.34)
Test 2	DHW	0.08 (0.04, 0.12)	0.13 (0.08, 0.16)	0.22 (0.12, 0.31)
	NHW	0.04 (0.01, 0.07)	0.03 (0.01, 0.05)	0.07 (0.02, 0.11)
	CHW	0.32 (0.26, 0.36)	0.48 (0.38, 0.55)	0.27 (0.20, 0.34)
Test 3	DHW	0.09 (0.05, 0.14)	0.15 (0.06, 0.21)	0.23 (0.11, 0.32)
	NHW	0.04 (0.00, 0.07)	0.05 (0.02, 0.08)	0.08 (0.02, 0.14)
	CHW	0.29 (0.23, 0.33)	0.45 (0.33, 0.51)	0.27 (0.17, 0.32)
Test 4	DHW	0.10 (0.06, 0.13)	0.14 (0.05, 0.20)	0.23 (0.11, 0.32)
	NHW	0.04 (0.01, 0.08)	0.04 (0.02, 0.05)	0.11 (0.01, 0.17)
	CHW	0.31 (0.25, 0.35)	0.47 (0.37, 0.52)	0.28 (0.17, 0.35)
Test 5	DHW	0.10 (0.06, 0.13)	0.14 (0.07, 0.18)	0.22 (0.11, 0.30)
	NHW	0.04 (0.01, 0.08)	0.04 (0.02, 0.05)	0.12 (0.05, 0.17)
	CHW	0.31 (0.25, 0.35)	0.47 (0.37, 0.51)	0.28 (0.16, 0.34)

Notes: *Test 1:* Using a natural cubic B spline function instead of a natural spline function to define the cross-basis function regarding exposure-response dimension. *Test 2*: Using three internal knots at equally spaced percentiles in the log scale instead of two internal knots at the 10th and 50th percentiles to define the knots for the cross-basis functions regarding exposure-response dimension. *Test 3*: Using four internal knots at equally spaced percentiles in the log scale instead of two internal knots at the 10th and 50th percentiles to define the knots for the cross-basis functions regarding exposure-response dimension. *Test 4 and 5*: Using four and five degrees of freedom (*dfs*) to capture the nonlinear effects of humidity instead of three *dfs*, respectively.

DHW=daytime heatwave; NHW=nighttime heatwave; CHW=day-night compound heatwave; NAD=non-accidental death; CVD=cardiovascular disease; RD=respiratory disease.

Supplementary Table 5. The pooled relative risks of daily non-accidental, cardiovascular, and respiratory mortality associated with daytime, nighttime, and day-night compound heatwave across East Asia, under different thresholds, durations, and the form of heatwave.

Heatwave	Types	NAD	CVD	RD
Changing Threshold *(from 90th to 92.5th, 95th, and 97.5th, respectively)*				
$HWT_{92.5}\_D_2$				
	DHW	1.11 (1.07, 1.15)	1.06 (1.04, 1.09)	1.07 (1.02, 1.12)
	NHW	1.03 (1.02, 1.05)	0.99 (0.95, 1.04)	1.03 (1.00, 1.05)
	CHW	1.21 (1.14, 1.29)	1.32 (1.21, 1.45)	1.12 (1.08, 1.17)
$HWT_{95}\_D_2$				
	DHW	1.03 (1.00, 1.06)	1.12 (1.04, 1.20)	1.01 (0.92, 1.12)
	NHW	1.02 (0.97, 1.07)	1.13 (1.04, 1.22)	0.98 (0.94, 1.03)
	CHW	1.26 (1.20, 1.32)	1.44 (1.30, 1.59)	1.21 (1.16, 1.26)
$HWT_{97.5}\_D_2$				
	DHW	1.00 (0.95, 1.04)	1.06 (0.93, 1.22)	1.04 (0.88, 1.23)
	NHW	1.04 (1.00, 1.08)	1.05 (0.96, 1.15)	1.05 (0.96, 1.15)
	CHW	1.32 (1.20, 1.45)	1.57 (1.41, 1.74)	1.18 (1.07, 1.30)
Changing Duration *(from at least 2 days to 3 and 4 days, respectively)*				
$HWT_{90}\_D_3$				
	DHW	1.01 (0.96, 1.06)	1.04 (0.96, 1.12)	0.99 (0.95, 1.03)
	NHW	1.01 (1.01, 1.02)	0.97 (0.94, 0.99)	1.01 (0.98, 1.04)
	CHW	1.22 (1.16, 1.29)	1.38 (1.26, 1.52)	1.25 (1.14, 1.38)
$HWT_{90}\_D_4$				
	DHW	1.07 (1.04, 1.11)	1.08 (0.98, 1.19)	0.86 (0.73, 1.02)
	NHW	1.03 (1.02, 1.05)	0.96 (0.93, 1.00)	0.98 (0.94, 1.02)
	CHW	1.27 (1.21, 1.34)	1.49 (1.39, 1.60)	1.23 (1.09, 1.39)
Changing Forms of heatwaves *(from CEHWI to a 0-1 variable)*				
$HWT_{90}\_D_2^*$				
	DHW	1.03 (1.03, 1.04)	1.04 (1.02, 1.06)	1.05 (1.01, 1.08)
	NHW	1.01 (1.00, 1.02)	0.99 (0.96, 1.02)	1.02 (1.00, 1.03)
	CHW	1.13 (1.10, 1.16)	1.18 (1.16, 1.21)	1.10 (1.08, 1.11)

Note: DHW=daytime heatwave; NHW=nighttime heatwave; CHW=day-night compound heatwave; NAD=non-accidental death; CVD=cardiovascular disease; RD=respiratory disease.
