# Supplementary material for: Nonlinear exposure-response associations of daytime, nighttime, and day-night compound heatwaves with mortality amid climate change
Source: Nat Commun. 2025 Jan 14;16:635. doi: 10.1038/s41467-025-56067-7 (PMC11729900; doi:10.1038/s41467-025-56067-7)
Supplement: Supplementary file 3 — Reporting Summary [file 41467_2025_56067_MOESM3_ESM.pdf]

nature portfolio

Corresponding author(s): Haidong Kan and Renjie Chen

Last updated by author(s): Dec 4, 2024

Reporting Summary

Nature Portfolio wishes to improve the reproducibility of the work that we publish. This form provides structure for consistency and transparency in reporting. For further information on Nature Portfolio policies, see our Editorial Policies and the Editorial Policy Checklist.

Statistics

For all statistical analyses, confirm that the following items are present in the figure legend, table legend, main text, or Methods section.

n/a	Confirmed	
☐	☒	The exact sample size (*n*) for each experimental group/condition, given as a discrete number and unit of measurement
☐	☒	A statement on whether measurements were taken from distinct samples or whether the same sample was measured repeatedly
☐	☒	The statistical test(s) used AND whether they are one- or two-sided<br>*Only common tests should be described solely by name; describe more complex techniques in the Methods section.*
☐	☒	A description of all covariates tested
☒	☐	A description of any assumptions or corrections, such as tests of normality and adjustment for multiple comparisons
☐	☒	A full description of the statistical parameters including central tendency (e.g. means) or other basic estimates (e.g. regression coefficient) AND variation (e.g. standard deviation) or associated estimates of uncertainty (e.g. confidence intervals)
☐	☒	For null hypothesis testing, the test statistic (e.g. *F*, *t*, *r*) with confidence intervals, effect sizes, degrees of freedom and *P* value noted<br>*Give P values as exact values whenever suitable.*
☒	☐	For Bayesian analysis, information on the choice of priors and Markov chain Monte Carlo settings
☒	☐	For hierarchical and complex designs, identification of the appropriate level for tests and full reporting of outcomes
☒	☐	Estimates of effect sizes (e.g. Cohen's *d*, Pearson's *r*), indicating how they were calculated

Our web collection on statistics for biologists contains articles on many of the points above.

Software and code

Policy information about availability of computer code

Data collection	No software was used to collect the data
Data analysis	All statistical analyses were conducted in R (Version 4.0.3). The "dlnm" (version 2.4.7) package was utilized for the first-stage analysis, while the "mvmeta" (version 1.0.3) package was employed for the second-stage analysis. A two-tailed P value of <0.05 was considered statistically significant.<br>The code repository for the main statistical analysis is available at: https://github.com/Simon-JD-Liu/East_Asia_heatwave

For manuscripts utilizing custom algorithms or software that are central to the research but not yet described in published literature, software must be made available to editors and reviewers. We strongly encourage code deposition in a community repository (e.g. GitHub). See the Nature Portfolio guidelines for submitting code & software for further information.

Data

Policy information about availability of data

All manuscripts must include a data availability statement. This statement should provide the following information, where applicable:

- Accession codes, unique identifiers, or web links for publicly available datasets
- A description of any restrictions on data availability
- For clinical datasets or third party data, please ensure that the statement adheres to our policy

The hourly temperature observations for all selected cities in this study were extracted from the ERA5-Land database (https://cds.climate.copernicus.eu). Relative

humidity data was obtained from the Korean Meteorological Office (https://www.kma.go.kr/eng/index.jsp), the Japanese Meteorological Agency (https://www.jma.go.jp/jma/indexe.html), and the Chinese Meteorological Data Sharing Service System(https://data.cma.cn/en). Daily concentrations of PM10 and O3 were downloaded from the Korean Research Institute of Public Health, the Japanese National Institute for Environmental Studies (https://www.nies.go.jp/index-e.html), and the Environmental Monitoring Centre of China (https://www.cnemc.cn/en/). City-specific attributes were obtained from Statistics Korea (https://kostat.go.kr/ansk/), the Statistics Bureau of Japan (https://www.stat.go.jp/), the National Bureau of Statistics of China (https://www.stats.gov.cn/sj/ndsj/), and the Census and Statistics Department of Hong Kong (https://www.censtatd.gov.hk/). Due to restrictions imposed by the data owners, the daily mortality data for each city of this study cannot be publicly released. They are only available upon request from the corresponding author and other relevant authors. Source data are provided with this paper.

Research involving human participants, their data, or biological material

Policy information about studies with human participants or human data. See also policy information about sex, gender (identity/presentation), and sexual orientation and race, ethnicity and racism.

Reporting on sex and gender	N/A
Reporting on race, ethnicity, or other socially relevant groupings	N/A
Population characteristics	N/A
Recruitment	N/A
Ethics oversight	N/A

Note that full information on the approval of the study protocol must also be provided in the manuscript.

Field-specific reporting

Please select the one below that is the best fit for your research. If you are not sure, read the appropriate sections before making your selection.

☒ Life sciences ☐ Behavioural & social sciences ☐ Ecological, evolutionary & environmental sciences

For a reference copy of the document with all sections, see nature.com/documents/nr-reporting-summary-flat.pdf

Life sciences study design

All studies must disclose on these points even when the disclosure is negative.

Sample size	Sample size calculations are not applicable to this study, as it is an ecological time-series analysis using population-level data from all available cities within the study period. The sample size reflects the total number of observations in the dataset, which is determined by the temporal resolution (daily data) and the duration of the study period.<br>Our study incorporated mortality data from 28 cities across three East Asian countries, namely South Korea (6 cities), Japan (7 cities), and China (15 cities), covering the period from 1981 to 2010.
Data exclusions	No data were excluded.
Replication	Our findings are solely generated from the statistical analyses described in the methodology section. Using code, we repeated all the analyses and stratified analyses, and the results were consistent.
Randomization	There was on experimental design or group allocation involved in this study
Blinding	There was on experimental design or group allocation involved in this study

Reporting for specific materials, systems and methods

We require information from authors about some types of materials, experimental systems and methods used in many studies. Here, indicate whether each material, system or method listed is relevant to your study. If you are not sure if a list item applies to your research, read the appropriate section before selecting a response.

Materials & experimental systems

n/a	Involved in the study
☒	☐ Antibodies
☒	☐ Eukaryotic cell lines
☒	☐ Palaeontology and archaeology
☒	☐ Animals and other organisms
☒	☐ Clinical data
☒	☐ Dual use research of concern
☒	☐ Plants

Methods

n/a	Involved in the study
☒	☐ ChIP-seq
☒	☐ Flow cytometry
☒	☐ MRI-based neuroimaging

Plants

Seed stocks

Report on the source of all seed stocks or other plant material used. If applicable, state the seed stock centre and catalogue number. If plant specimens were collected from the field, describe the collection location, date and sampling procedures.

Novel plant genotypes

Describe the methods by which all novel plant genotypes were produced. This includes those generated by transgenic approaches, gene editing, chemical/radiation-based mutagenesis and hybridization. For transgenic lines, describe the transformation method, the number of independent lines analyzed and the generation upon which experiments were performed. For gene-edited lines, describe the editor used, the endogenous sequence targeted for editing, the targeting guide RNA sequence (if applicable) and how the editor was applied.

Authentication

Describe any authentication procedures for each seed stock used or novel genotype generated. Describe any experiments used to assess the effect of a mutation and, where applicable, how potential secondary effects (e.g. second site T-DNA insertions, mosiacism, off-target gene editing) were examined.
